# Low Self-Control: A Hidden Cause of Loneliness?

**DOI:** 10.1177/01461672211007228

**Published:** 2021-04-15

**Authors:** Olga Stavrova, Dongning Ren, Tila Pronk

**Affiliations:** 1 Tilburg University, Netherland

**Keywords:** self-control, loneliness, ostracism, social exclusion, daily diary, experience sampling

## Abstract

Loneliness has been associated with multiple negative outcomes. But what contributes to loneliness in the first place? Drawing from the literature on the importance of self-regulatory ability for successful social functioning, the present research explored the role of low self-control as a factor leading to loneliness. A set of four studies (and three additional studies in Supplementary Online Materials) using cross-sectional, experimental, daily diary, and experience sampling methods showed that lower self-control is associated with higher loneliness at both trait and state levels. Why does low self-control contribute to loneliness? Self-control failures that have negative implications for others lead to higher risks for being ostracized by others, which predicts increased feelings of loneliness over time. These results suggest that low self-control, which is often associated with negative intrapersonal outcomes, can have important interpersonal consequences by evoking ostracism, and consequently, loneliness.

Loneliness is prevalent. Up to two thirds of Americans experience moderate-to-severe loneliness (Musich et al., 2015) and about one third of Britons report feeling lonely often or very often (BBC, 2018). Loneliness describes a perceived gap between the desired and the experienced amount of intimacy, connectedness, and closeness with others (Hawkley et al., 2010). It is well-established that loneliness has negative consequences for well-being, leading to cognitive decline (James et al., 2011), depression (Holvast et al., 2015), and premature mortality (Steptoe et al., 2013). Given the detrimental effects of loneliness, it is important to understand what contributes to loneliness in the first place. While research on the consequences of loneliness has been prolific, the antecedents of loneliness have received much less empirical attention. In addition, the existing studies on the antecedents of loneliness are limited in several ways, such as the use of correlational data that preclude causal inferences, and the focus on specific populations (e.g., children and adolescents, older adults, patients) that puts constrains on the findings’ generalizability (for a review, see Cohen-Mansfield et al., 2016).

The present research was designed to advance our understanding of the antecedents of loneliness. Drawing from the literature on the importance of self-regulation for interpersonal success (Baumeister & Exline, 1999; Tangney et al., 2004), the present investigation focused on self-control. Using survey, experimental and intensive longitudinal methods, we explored the role of self-control as a factor contributing to higher loneliness.

## Antecedents of Loneliness

Studies on the antecedents of loneliness showed that loneliness might have roots in socially undesirable traits and behaviors, including disagreeableness, poor social skills and antisocial behavioral tendencies. For example, less agreeable adolescents are more likely to report a chronically high level of loneliness (Vanhalst et al., 2013), and decreases in agreeableness are associated with increases in loneliness over time (Mund & Neyer, 2016).

Poor socio-emotional ability has been associated with higher loneliness too. Adolescents with lower emotional intelligence are more likely to become lonely over time (Wols et al., 2015), and young adults who report poorer social skills tend to experience higher levels of loneliness (DiTommaso et al., 2003). Similarly, elementary school children who were rated as more cooperative, less snobbish and less aggressive by their teachers were more liked by their peers and reported lower level of loneliness (Mouratidis & Sideridis, 2009).

## The Role of Self-Control

Besides traits and behaviors that fall in the agreeableness spectrum, researchers are now increasingly recognizing the importance of self-regulatory ability or self-control in promoting normative and socially desirable behaviors (Baumeister & Exline, 1999; Baumeister & Vohs, 2007; DeBono et al., 2011; Stavrova & Kokkoris, 2017). Self-control can be defined as the ability to prioritize long-term goals over tempting desires, urges, and impulses (de Ridder et al., 2018; Milyavskaya et al., 2019). Some streams of literature have focused on self-control as a trait, showing that some individuals are routinely better at solving self-control dilemmas than others (Tangney et al., 2004). Other research studied state self-control, focusing primarily on momentary states of low self-control as a result of effortful inhibition tasks (e.g., Stroop task or ego depletion paradigms). Interestingly, individuals scoring high on trait self-control do not necessarily do better on effortful inhibition tasks (Duckworth & Kern, 2011; Imhoff et al., 2014). This lack of consistent associations between trait self-control and proneness to depletion effects gave rise to a call for a stronger conceptual and empirical integration of research on trait and state self-control (de Ridder et al., 2018). Herein, we adopt the trait and state approach to personality that defines personality states as behavioral manifestations of the respective personality traits (Fleeson, 2001). Following this approach, we define state self-control as momentary experiences of self-control failures/success, that is, giving into/not giving into a tempting momentary desire at the cost of a long-term goal. We explore the role of both trait and state self-control in individuals’ experience of loneliness.

Why would lower self-control (as a trait or as a state) contribute to loneliness? We propose that the risk of being ostracized might be one of the mechanisms linking low self-control to higher loneliness. Even though, by definition, high self-control is primarily associated with positive outcomes for the self (i.e., successful goal pursuit), it has been shown to have some beneficial side-effects for others. High self-control facilitates overcoming egoistic or antisocial impulses for the sake of the group’s interests (Baumeister & Exline, 1999). In fact, evolutionary theories of self-control even suggest that it has evolved as a way to manage a conflict between selfish desires and cultural norms (e.g., norms of prosocial behavior) (Baumeister & Vohs, 2007). Indeed, empirical studies demonstrated that self-control facilitates behaviors that strengthen social bonds, such as norm-compliance (DeBono et al., 2011), cooperative behaviors (Kocher et al., 2017), self-sacrificing in romantic relationships (Findley et al., 2014), and reduced likelihood of aggressive responses (Denson et al., 2012).

Consistent with these findings, high self-control has positive reputational consequences. For example, perceptions of self-control in others are associated with perceptions of trustworthiness and likeability (Buyukcan-Tetik et al., 2015; Liu et al., 2017). Individuals who score higher on self-control are attributed a higher level of trustworthiness and morality more generally (Betts & Rotenberg, 2007; Marr et al., 2019; Righetti & Finkenauer, 2011). Individuals engaging in self-control failures—even when the failures have no obvious negative consequences for others (e.g., overeating or overspending)—are perceived as untrustworthy (Righetti & Finkenauer, 2011).

One of the common claims in trust and trustworthiness research is that people trust others because they perceive them as benevolent (i.e., warm, caring) or able (i.e., competent and skilled) (Mayer et al., 1995). Hence, people might infer low trustworthiness from self-control failures because they see these failures as a cue that the target individual lacks the natural predisposition to behave prosocially (e.g., being coldhearted and uncaring) or the ability to do so. Both perceptions might elicit ostracism intentions (Rudert et al., 2020). Since low self-control has been associated with both, low prosocial disposition (e.g., agreeableness) and norm violations (DeBono et al., 2011; Stavrova & Kokkoris, 2017), we explored whether low self-control elicits ostracism due to perceptions of a lack of prosocial disposition or inability to follow cultural norms, including norms prohibiting other-harming (e.g., free-riding) behaviors.

While the present research focuses on the effect of self-control on loneliness, experience of loneliness might undermine self-control as well, resulting in a vicious cycle. It has been suggested that the experience of social exclusion might deprive individuals from the resources needed to successfully engage in self-control tasks (Campbell et al., 2006). Experiences of exclusion have indeed been shown to interfere with executive control functions (Baumeister et al., 2005; Campbell et al., 2006); chronic ostracism has been associated with lower self-control in adolescents (DeWall et al., 2012); and experimental manipulations of exclusion were linked to behaviors indicative of self-control failures, such as unhealthy food consumption (Baumeister et al., 2005; Burson et al., 2012). Building on this past research, here we explored the potentially bidirectional effects of loneliness and self-control.

## The Present Research

Study 1 examined the trait-level associations between self-control and loneliness in a large nationally representative data set. Study 2’s weeklong daily diary study explored the effect of both trait and state self-control on daily loneliness and tested the prospective bidirectional associations between daily experiences of self-control failures and daily loneliness. To test the role of ostracism, Study 3 used experimental methods to examine whether individuals are more willing to ostracize low (vs. high) self-control others. Finally, Study 4’s experience sampling (ESM) study tested the mediating role of ostracism in the association between self-control failures and loneliness in a longitudinal mediation analysis. This final study also explored whether self-control failures are associated with ostracism and loneliness only when they bring about negative consequences for others.

One additional study (reported in SOM) showed that individuals anticipate ostracism and loneliness following their own public self-control failures; and two additional studies explored an alternative mechanism of the effect of low control on loneliness: self-control failures may lead to stronger intention to withdraw from social interactions, which in turn lead to higher loneliness. No evidence was obtained for this alternative mechanism (see SOM for details).

The data, study materials, and the analyses scripts can be downloaded at https://osf.io/3yvp2/.

## Study 1

The negative association between trait self-control and loneliness has been previously detected in samples of students and children (Hamama et al., 2000; Özdemir et al., 2014). Herein, we extended these findings by estimating this association using large nationally representative data. To control for potential confounds, such as trait agreeableness and other Big Five traits (that have been associated with loneliness in previous research; Mund & Neyer, 2016), we included the Big Five scores as control variables. The data can be accessed at the study’s website: https://www.lissdata.nl/access-data.

### Method

#### Participants

We used the data from the Longitudinal Internet Studies for the Social Sciences (LISS, 2015) in the Netherlands. LISS is a nationally representative panel study that surveys about 7,000 individuals annually since 2007. Panel members are asked to complete different questionnaires throughout the year. In February 2012, participants completed a measure of self-control (as part of “Proactive coping and health behavior” questionnaire) and loneliness (as part of “Social integration and leisure” questionnaire). Individuals who completed both measures constituted our sample: 2,701 individuals (aged between 19 and 90, *M*_age_ = 52.02, *SD*_age_ = 16.33, 52.4% male).

#### Measures

Participants’ self-control was measured with the Brief Trait Self-control Scale (Tangney et al., 2004) (13 items, sample item “I am good at resisting temptation,” Cronbach’s α = .78). Responses were given on a 5-point scale (1 = completely not applicable, 5 = completely applicable).

To measure loneliness, a 6-item version of the UCLA loneliness scale (Russell, 1996) was included in the study (sample items: “I miss having people around me” and “I have a sense of emptiness around me”). Participants indicated whether each statement applies to them (1 = *yes*, 2 = *no*, 3 = *don’t know/don’t want to say*). Participants’ responses were recoded such that higher values indicate more loneliness (and “*don’t know/don’t want to say*” is coded as missing) and averaged into an index of loneliness (Cronbach’s α = .78).

To measure the Big Five (agreeableness, extraversion, conscientiousness, emotional stability, and openness), the LISS used the 50-item set of the International Personality Item Pool (1 = *very inaccurate*, 5 = very accurate) (Goldberg, 1992). All scales showed adequate to good reliability (Cronbach’s αs between .76 and .88). As LISS used a planned missingness design, 21% of the participants completed the Big Five scales in February 2012, while for the remaining 79%, we took the Big Five scores from the year before (2011).

We also included socio-demographic control variables that have been associated with loneliness and self-control in past studies (Nakhaie et al., 2000; Pinquart & Sorensen, 2001): gender (1 = *male*, 0 = *female*), age, whether the participant had a live-in partner (1 = *yes*, 0 = *no*), the number of children in the household (0–6), education (three categories: high school, college, and university), employment status (five categories: employed, unemployed, student, housekeeper, and other), and household income before taxes in Euros.

### Results

Table 1 shows the means, standard deviations, and zero-order correlations among the variables. As expected, lower self-control was associated with higher loneliness (*r* = −.15, *p* < .001, 95% confidence interval [CI] = [−.19; −.11]).

**Table 1.** Means, Standard Deviations, and Correlations, Study 1.

Variable	*M*	*SD*	1	2	3	4	5	6	7	8
1. Self-control	3.42	0.57	—	—	—	—	—	—	—	—
2. Loneliness	0.10	0.20	−.15*** [−.19, −.11]	—	—	—	—	—	—	—
3. Agreeableness	3.87	0.49	.16*** [.12, .20]	−.12*** [−.16, −.08]	—	—	—	—	—	—
4. Conscientiousness	3.74	0.52	.48*** [.45, .51]	−.09*** [−.13, −.05]	.32*** [.29, .36]	—	—	—	—	—
5. Openness	3.47	0.50	.08*** [.04, .11]	−.03 [−.07, .01]	.25*** [.22, .29]	.24*** [.21, .28]	—	—	—	—
6. Emotional stability	3.50	0.67	.30*** [.26, .33]	−.27*** [−.31, −.24]	.03 [−.01, .07]	.20*** [.16, .24]	.19*** [.16, .23]	—	—	—
7. Extraversion	3.27	0.63	.08*** [.04, .11]	−.17*** [−.21, −.13]	.29*** [.26, .33]	.12*** [.08, .16]	.34*** [.31, .37]	.25*** [.21, .28]	—	—
8. Age	52.02	16.33	.30*** [.27, .34]	−.07*** [−.11, −.03]	.05* [.01, .08]	.14*** [.11, .18]	−.14*** [−.18, −.10]	.11*** [.08, .15]	−.07*** [−.11, −.03]	—
9. Income	4,391.39	6,905.42	−.00 [−.04, .04]	−.04* [−.08, −.00]	−.01 [−.05, .03]	−.01 [−.05, .03]	.04* [.00, .08]	.03 [−.01, .07]	.04 [−.00, .08]	−.04* [−.08, −.01]

*Note.* Values in square brackets indicate the 95% confidence interval for each correlation.

* *p* < .05. **p < .01. ***p < .001.

To account for nonindependence in the data (participants nested within households), we used multilevel regression models. We specified a random intercept at the level of households. The results are presented in Table 2. Model 1 tested the zero-order effect of self-control on loneliness. Model 2 added the Big Five scores and the socio-demographic and economic characteristics. Confirming the zero-order correlations, lower self-control predicted higher loneliness (β = −.15, *p* < .001, 95% CI = [−.18; −.11]). This effect was robust, although reduced in size (β = −.07, *p* = .002, 95% CI [−.12; −.03]), when we added the Big Five and socio-demographic controls in Model 2. The effect of self-control was similar in size to the effects of previously identified predictors of loneliness: agreeableness (β = −.09, *p* < .001, 95% CI [−.14; −.05]) and extraversion (β = −.11, *p* < .001, 95% CI [−.16; −.07]), but much smaller than the strongest predictor of loneliness in this study: emotional stability (β = −.24, *p* < .001, 95% CI [−.28; −.20]). It is noteworthy that, conscientiousness, the trait that is most conceptually related to self-control, was associated with loneliness at the level of zero-order correlations (*r* = −.09, *p* < .001, 95% CI [−.13; −.05]), but did not predict loneliness in the multiple regression analysis (β =.02, *p* = .304, 95% CI [−.01; .08]). These results (and additional analyses in SOM) suggested that the zero-order association between conscientiousness and loneliness were likely due to variance overlap with trait self-control and other big five (extraversion, agreeableness, and emotional stability).

**Table 2.** Multilevel Regression Analyses, Study 1.

	DV: Loneliness			
	Model 1		Model 2	
Predictor	*B*	*SE*	*b*	*SE*
Self-control	−.15***	.02	−.07**	.02
Openness	—	—	.06**	.02
Conscientiousness	—	—	.03	.02
Extraversion	—	—	−.11***	.02
Agreeableness	—	—	−.09***	.02
Emotional stability	—	—	−.24***	.02
Male	—	—	.02	.04
Age	—	—	−.003	.002
Partner	—	—	−.17***	.05
Number of children	—	—	−.0008	.02
Employment status: Unemployed	—	—	.27*	.13
Employment status: Student	—	—	−.02	.10
Employment status: Housekeeper	—	—	.13	.08
Employment status: Other	—	—	.01	.06
Education: College	—	—	.009	.04
Education: University	—	—	.01	.08
Income	—	—	−.000003	.000002

*Note. b* = unstandardized regression coefficients. Reference category for Employment status: Employed; for Education: High school.

* *p* < .05. **p < .01. ***p < .001.

### Discussion

Study 1 provided first evidence of the negative association between trait self-control and loneliness in a large nationally representative sample in the Netherlands. This association was robust against controlling for the Big Five personality traits and comparable in size to the effects of the traits (e.g., agreeableness and extraversion) previously identified as important predictors of loneliness (Mund & Neyer, 2016).

## Study 2

Study 2 examined the associations between self-control and loneliness in daily life. Using intensive longitudinal methods (daily diary), we explored how both trait and state self-control (daily self-control failures) are related to daily experiences of loneliness. In addition, making use of the longitudinal data structure, we tested the prospective effect of self-control on loneliness by exploring whether self-control failure on one day is associated with more loneliness on the following day (and another way around).

### Participants

We recruited 536 participants on Amazon Mechanical Turk (MTurk) to participate in a 7-day-long diary study. Thirty did not pass an attention check (the same as in Studies 2 and 3) and were removed. Of the remaining participants, 460 completed at least one daily assessment and constituted our final sample (52.6% male, *M*_age_ = 36.45, *SD*_age_ = 11.60). Participants completed 5.56 daily assessments (*SD* = 1.88), on average (see SOM for power analysis).

### Procedure and Measures

Participants were first invited to take part in an intake survey that included a measure of *trait self-control*. We used the Brief Self-control Scale (Tangney et al., 2004) (13 items, e.g., “People would say that I have iron self-discipline”). Responses were given on a 5-point scale ranging from “*not at all*” to “*very much*” (Cronbach’s α = .89).

To make sure that the obtained estimate of the association between self-control and loneliness is not inflated due to confounding with other factors, we included a number of variables in our analyses as covariates: life satisfaction, presence of meaning, and search for meaning—factors that have been linked with both self-control and loneliness in past research (e.g., Stavrova et al., 2018). Participants overall judgment of *life satisfaction* was measured with the following item: “Taking all things together, how satisfied are you with your life as a whole?” (1 = *extremely dissatisfied*; 10 = *extremely satisfied*). The *presence of meaning* and *search for meaning* were measured using items from the Meaning in Life Questionnaire (Steger et al., 2006) (presence: 5 items, e.g., “My life has a satisfying sense of purpose,” Cronbach’s α = .96; search: 5 items, e.g., “I’m seeking a purpose or mission for my life,” Cronbach’s α = .95).

Participants who completed the intake survey were invited to participate in the daily diary part of the study that started the next day. To allow participants to complete the daily assessments, they were sent an online link to each daily survey. The link was sent at 4 p.m. Eastern Standard Time and was active for 24 hours. Most participants completed daily assessments within 3.62 (*SD* = 4.76) hours after the invitation letter was sent. The study continued for the period of 7 days.

To measure *daily loneliness*, participants indicated to what extent they felt lonely in the past 24 hours. To measure *daily self-control failure*, participants indicated whether, in the past 24 hours, they gave in to a temptation. Daily measures additionally included *daily meaning* (“felt that your life was meaningful”), *daily happiness* (“felt happy”), *daily sadness* (“felt sad”), *daily self-esteem* (“felt pretty good about yourself”), and *daily sense of true self* (“felt as if you know yourself very well” and “felt like you were really being yourself,” averaged, average *r* = .28). All daily measures used a 7-point scale (1 = *not at all*, 7 = *a lot*).

### Results

Trait self-control was negatively associated with the average experience of loneliness across the 7 days (*r* = −.37, *p* < .001, 95% CI = [−.45; −.29]); reports of self-control failures were positively associated with daily reports of loneliness (*r* = .24, *p* < .001, 95% CI = [.16; .33]) (Supplemental Table S1).

#### Trait self-control and daily loneliness

To account for the nonindependence of observations (daily assessments nested within individuals), we estimated the effect of trait self-control on daily loneliness using multilevel regression. The models included a random intercept at the level of participants; to account for longitudinal data structure, we additionally specified an error structure that allowed for correlations between adjacent time points for the same participant (Finch et al., 2019). Model 1 (see Table 3) showed that individuals with lower trait self-control experienced more loneliness within the observation period (7 days; *b* = −0.75, *p* < .001, 95% CI = [−0.92; −0.58]). Model 2 included all control variables listed above (life satisfaction, presence, and search for meaning in life; daily happiness, daily sadness, daily self-control, and daily true self). The effect of trait self-control remained significant (*b* = −0.24, *p* < .001, 95% CI = [−0.36; −0.11]).

**Table 3.** Effects of Trait Self-Control and Daily Self-Control Failures on Daily Loneliness (Contemporaneous Effects), Study 2.

	DV: daily loneliness							
	Model 1		Model 2		Model 3		Model 4	
Predictor	*b*	*Se*	*B*	*se*	*b*	*se*	*b*	*se*
Trait self-control	−.75***	.09	−.24***	.06	—	—	—	—
Daily self-control failures	—	—	—	—	.03*	.02	.04*	.02
Life satisfaction	—	—	−.05*	.02	—	—	−.24***	.03
Presence of meaning	—	—	.13*	.06	—	—	.05	.09
Search for meaning	—	—	.13***	.03	—	—	.23***	.04
Daily happiness	—	—	−.08***	.02	—	—	−.09***	.02
Daily sadness	—	—	.41***	.02	—	—	.34***	.02
Daily meaning	—	—	−.02	.02	—	—	−.03*	.02
Daily self-esteem	—	—	−.06**	.02	—	—	−.09***	.02
Daily true self	—	—	−.11***	.03	—	—	−.06*	.03

*Note. b* = unstandardized regression coefficients. Daily predictors in Models 3 and 4 were centered within-persons.

* *p* ≤ .05. **p < .01. ***p < .001.

#### Daily self-control failures and daily loneliness: Contemporaneous associations

To test whether individuals experience more loneliness on days where they reported self-control failures, we regressed daily loneliness on daily self-control failures. We used multilevel regression with the same setup as described above. As we were interested in the associations between constructs measured within individuals, we centered daily predictors within-persons (Enders & Tofighi, 2007). Model 1 (Table 3) showed that daily self-control failures were positively associated with daily loneliness: although this effect was on the boundary of conventional level of significance without covariates (*b* = 0.03, *p* = .053, 95% CI = [−0.0004; 0.07]), it was robust against controlling for the covariates listed above (*b* = 0.04, *p* = .019, 95% CI = [0.01; 0.07]).

#### Daily self-control failures and daily loneliness: Prospective effects

Next, we made use of the longitudinal nature of the data to examine whether experiencing self-control failures prospectively affects the experience of loneliness and another way around. To test whether self-control failure prospectively predicts loneliness, we regressed loneliness at day *t* on self-control failures on day *t*−1 and loneliness on day *t−*1. This way, we could examine whether experiencing self-control failure on any specific day leads to an increase in loneliness the following day. Second, to test whether the feeling of loneliness prospectively predicts self-control failures, we regressed self-control failures on day *t* on loneliness on day *t−*1 and self-control failures on day *t−*1. In both cases, we relied on the multilevel regression method, as describe above; all daily predictors were centered within-persons. The results are shown in Table 4. The prospective effect of self-control failures on loneliness was significant: experiencing self-control failures on one day predicted feeling lonely on the following day (*b* = 0.06, *p* < .001, 95% CI = [0.03; 0.10]); whereas the prospective effect of loneliness on self-control failures did not reach significance (*b* = −0.01, *p* = .70, 95% CI = [−0.07; 0.05]). These results were not affected by the control variables (see Table 4).

**Table 4.** Daily Self-Control Failures and Daily Loneliness, Prospective Effects, Study 2.

	DV: daily loneliness *t*				DV: daily self-control failures *t*			
	Model 1		Model 2		Model 1		Model 2	
Predictor	*b*	*SE*	*B*	*SE*	*b*	*SE*	*b*	*SE*
Daily self-control failures *t*−1	.06***	.02	.06**	.02	−.32***	.02	−.32***	.02
Daily loneliness *t*−1	−.22***	.02	−.24***	.03	−.01	.03	−.04	.03
Life satisfaction	—	—	−.24***	.03	—	—	−.13**	.04
Presence of meaning	—	—	.06	.09	—	—	.27**	.10
Search for meaning	—	—	.22***	.04	—	—	−.03	.05
Daily happiness *t*−1	—	—	.01	.03	—	—	.03	.03
Daily sadness *t*−1	—	—	.05*	.02	—	—	.05	.03
Daily meaning *t*−1	—	—	.05*	.02	—	—	−.01	.02
Daily self-esteem *t*−1	—	—	.03	.02	—	—	−.02	.03
Daily true self *t*−1	—	—	−.07*	.03	—	—	−.04	.04

*Note. b* = unstandardized regression coefficients. Daily predictors were centered within-persons. Note that lagged effects of both loneliness and self-control failures are negative, suggesting that higher values on one day are associated with lower values on the following day; since daily values were centered within-persons, we assume that this is a result of the “regression to the mean” effect (using noncentered values result in positive autoregressive effects; note that the cross-lagged effects are significant regardless of what centering is used).

* *p* < .05. **p < .01. ***p < .001.

##### Trait and state self-control

**Figure 1.**
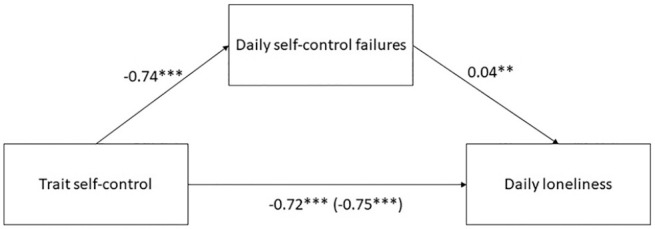
Mediation analysis, Study 2. *Note.* Unstandardized regression coefficients. Indirect effect: −0.03, 95% CI = [−0.06; −0.01]. The number inside the brackets is the total effect (c), the number outside the brackets is the direct effect (c’). *p < .05. **p < .01. ***p < .001.

Given the ambiguity regarding the interrelations of trait and state self-control in previous studies (for an overview, see de Ridder et al., 2018), we explored the associations between trait and state self-control in the present data. Trait self-control was significantly associated with less daily self-control failures (*b* = −0.74, *p* < .001, 95% CI [−0.92; −0.58]). Next, we examined whether the effect of trait self-control on daily loneliness is mediated by daily self-control failures. To assess path “a” of the mediation model, we regressed trait self-control on daily self-control failures; to assess path “b,” we regressed daily self-control failures on daily loneliness (while controlling for trait self-control). In both cases, we used multilevel regression with a random intercept for participants and autoregressive error structure (as described above; predictors were not centered). We used Monte Carlo simulation to estimate the significance of the indirect effect (a*b). The indirect effect was significant (−0.03, 95% CI = [−.06; −.01]), providing evidence for mediation (see Figure 1).

### Discussion

Study 2 explored the interplay of self-control and loneliness in daily life. It showed that individuals with higher trait self-control tended to experience less loneliness on a day-to-day basis and that daily failures at resisting temptations were associated with more daily loneliness. High trait self-control individuals were less likely to give in to temptations on a daily basis and this partially explained their lower daily loneliness. Longitudinal analyses confirmed the prospective effect of daily self-control failures on daily loneliness, but not the reverse.

## Study 3

Why is low self-control associated with more loneliness? We proposed that low self-control signals lower trustworthiness and thus leads to an increased likelihood of ostracism and, consequently, loneliness. Study 3 explored this possibility by testing whether people display stronger ostracism intentions toward others who show signs of low self-control (i.e., commit self-control failures). In addition, we explored potential mediators of this effect: perception of target prosocial disposition versus ability to follow prosocial norms.

Participants were randomly assigned to read a description of a person who displays either low or high self-control and to report their ostracism intentions toward that person, as well as their perception of that person’s prosocial disposition and ability to forgo self-interest and follow prosocial norms. We expected participants to be more willing to ostracize a low than a high self-control target. Our hypotheses, procedures, and analysis plans were preregistered (https://aspredicted.org/blind.php?x=82mh6z). There were no deviations from the preregistered plans.

### Method

#### Participants

A power analysis using g*power (Faul et al., 2009) indicated that to detect a small effect (*d* = 0.35; 80% power, α = 5%, two-tailed test, independent-sample *t*-test), we would need 200 participants. To compensate for participants failing the attention check, we recruited 230 participants on Amazon Mechanical Turk (MTurk). Two hundred thirty-two participants completed the survey. Of those, six failed an attention check question (s. below), resulting in the final sample of 226 individuals (*M*_age_ = 35.92, *SD*_age_ = 11.25, 56.4% male).

#### Procedure and measures

Participants learned that they will make several judgments about Robin, an MTurk worker. Robin was described as follows: “30 year old, works full-time and has a three-year old daughter.

Robin took part in one of our previous studies where we asked participants to describe an event that had happened to them in the previous week.” Participants were then shown Robin’s report:

Lately I have some money problems . . . it’s not that I’m in debt, but I definitely need to save some money for my further education which is really important for me! Last Wednesday I was around the city having a walk, and I ended up in my favorite electronics store.

In the low self-control condition, the text ended with: “I was having a look at all the cool smartphones and tablets available and at the new entries, and I ended up buying a new smartphone (even though I already had one).” In the high self-control condition, ended with: “I was having a look at all the cool smartphones and tablets available and at the new entries, but eventually I did not buy anything” (the manipulation adapted from Righetti & Finkenauer, 2011).

To measure the dependent variable, participants were asked to imagine that Robin is a new colleague at their work and indicated how likely they would be to ostracize Robin using a seven-item *ostracism intention scale* (adapted from Hales et al., 2016; for example, “I might find myself excluding Robin”). Participants used a 7-point scale (1 = *strongly disagree*; 7 = *strongly agree*; Cronbach’s α = .91).

We used three items to measure *target perceived prosocial disposition*: “Robin cares about other people,” “Robin takes time for others,” and “Robin sympathizes with others’ feelings.” Another set of three items was used to measure *target perceived ability to follow prosocial norms*: “Robin has enough will power to not engage in behaviors that might hurt others,” “Robin is able to resist the temptation to behave selfishly,” and “Robin can easily follow the socially desirable and acceptable standards of behavior.” All items used a 7-point scale (1 = *strongly disagree*; 7 = *strongly agree*) and both scales showed good reliabilities (Cronbach’s α = .95 and .89, respectively; were correlated at *r* = .55, *p* < .001). The order in which perceived prosocial disposition and ability were answered was randomized across the participants, it did not influence the effect of the manipulation, *F*(4,219) = .13, *p* = .97.

Finally, participants responded to two manipulation check questions: “Robin is bad at resisting temptations (reverse-coded)” and “Robin has strong self-control” (7-point response scale, Cronbach’s α = .92), responded to an attention check question (“To monitor data quality, please select the middle of the scale here”), and provided basic socio-demographic information.

### Results

#### Manipulation check

Participants perceived the target in the self-control failure (vs. nonfailure) condition as having poorer self-control (*M* = 5.59, *SD* = 1.04 vs. *M* = 2.75, *SD* = 1.18), *t*(224) = 19.17, *p* < .001). Hence, the manipulation was successful.

#### Ostracism intentions

Participants showed a stronger intention to ostracize the target who failed at self-control (*M* = 2.61, *SD* = 1.22) than the target who did not (*M* = 2.06, *SD* = 0.99), *t*(213.449) = 3.77, *p* < .001, *d* = .50.

#### Prosocial disposition and ability to follow prosocial norms

Participants perceived the target who failed at resisting temptation (vs. did not fail) as having a weaker general predisposition for prosociality (*M* = 4.71, *SD* = 1.08 vs. *M* = 5.19, *SD* = 1.00) and a weaker ability (*M* = 3.87, *SD* = 1.05 vs. *M* = 5.63, *SD* = 0.96) to follow social norms of prosociality, *t*(224) = 3.46, *p* = .001, *d* = .79, and *t*(224) = 13.11, *p* < .001, *d* = .45. Also, perceptions of a weaker disposition and ability were associated with a stronger ostracism intention (*r*_disposition_ = −.36 and *r*_ability_ = −.48, both *p*s < .001).

#### Mediation analysis

**Figure 2.**
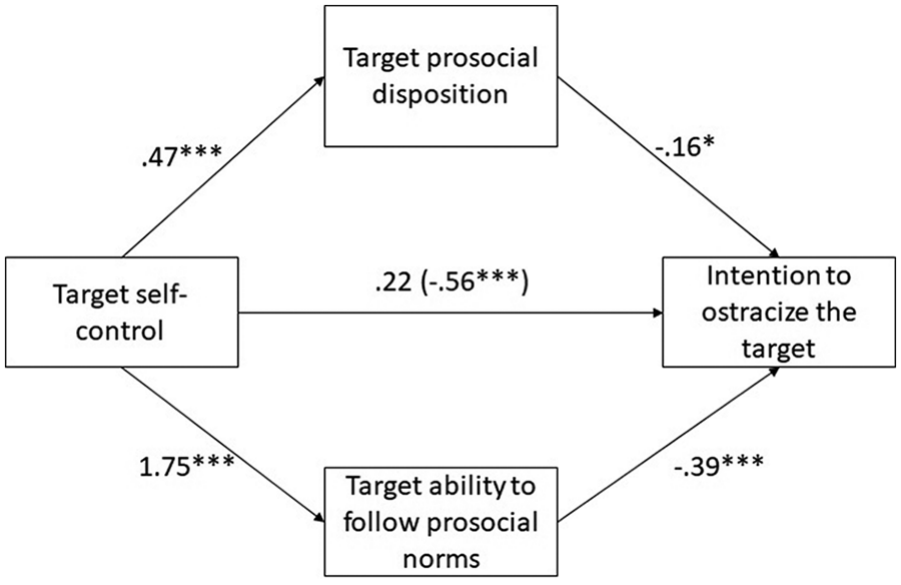
Mediation analyses, Study 3. *Note.* Unstandardized regression coefficients. Indirect effect via ability: −.70, 95% CI = [−1.02; −.40]; indirect effect via prosocial disposition: −.08, 95% CI = [−.18; .0002]. The number inside the brackets is the total effect (c), the number outside the brackets is the direct effect (c’). *p < .05, ** *p* < .01, *** *p* < .001.

We used the process macro v3.4 (Hayes, 2017) with a bootstrapping method with 20,000 re-samples to examine whether perceived prosocial disposition and ability of the target to follow prosocial norms mediate the effect of target self-control on participants’ willingness to ostracize the target. Disposition and ability were tested as parallel mediators. The results are presented on Figure 2. Test of indirect effects showed that the effect of self-control failure was significantly mediated by target perceived ability (−.70, 95% CI = [−1.02; −.40]), but not by perceived disposition (−.08, 95% CI = [−.18; .0002]). This suggests that the target low in self-control was attributed a poorer ability to follow prosocial norms than the target high in self-control; and perceiving weaker “prosociality ability” in the target was related to participants’ intention to ostracize the target.

### Discussion

Study 3 using experimental design demonstrated that self-control failures might increase the risk of ostracism. Participants showed stronger intentions to ostracize a target who failed at self-control than a target who did not fail. Although a low (vs. high) self-control target was seen as being low in both the general prosocial disposition and the ability to follow norms of prosociality, only the ability mediated the effect of target’s self-control on ostracism intentions of the target. This finding provides support to the theoretical assertion that people see others’ self-control lapses a cue to a lack of the ability to follow cultural norms, including norms prohibiting other-harming (e.g., free-riding) behaviors.

## Study 4

So far, we have shown that low self-control (trait and state) is associated with higher loneliness and that people show stronger intentions to ostracize low (vs. high) self-control others. But do low self-control individuals experience ostracism at a higher rate and does this experience explain their elevated feelings of loneliness? Study 4 was designed to answer these questions using ESM method. First, we expected low self-control to be associated with more perceived ostracism and loneliness. Second, we expected perceived ostracism to mediate the effect of low self-control on the experience of loneliness. We expected these effects to operate at both, trait and state level. Regarding the latter, making use of the longitudinal data structure, we expected perceived ostracism to act as a longitudinal mediator of the effect of state self-control (momentary self-control failures) on state loneliness.

In addition, we also took a closer look at different types of self-control failures and examined the importance of other-harming consequences (associated with self-control failures) in driving exclusion and loneliness. Specifically, we explored whether the effect of self-control failures on ostracism and loneliness depends on whether the failures are associated with negative or positive consequences for others.

### Participants

Participants were U.K. residents recruited on Prolific Academic. Four hundred fifty-three individuals completed the intake survey. Three hundred and eight participants correctly responded to an attention check question (see SOM) and were invited to take part in the 7-day-long ESM study. Two hundred seventy-two participants (88%) accepted the invitation and completed at least one assessment (note that of 272, seven participants could not be matched with the data from the intake survey due to entering a wrong iD). Measures of perceived ostracism and loneliness were included in all momentary assessments; measures of momentary self-control failures were included in the last 3 days of the ESM study (due to a technical error). Therefore, depending on the analysis, the final sample size ranged between 265 individuals/7,717 assessments (*M*_age_ = 34.33, *SD*_age_=12.47, 26% male) and 200 individuals/604 assessments (*M*_age_ = 34.01, *SD*_age_=12.13, 23% male).

### Procedure and Measures

#### Trait measures

To measures *trait self-control*, we used the Brief Self-control Scale (Tangney et al., 2004) (13 items, for example, “People would say that I have iron self-discipline”). Participants responded using a 5-point scale ranging from “*not at all*” to “*very much*” (Cronbach’s α = .86).

*Trait loneliness* was measured using the 20-item version of the UCLA scale (e.g., “I feel left out,” Cronbach’s α = .95). Responses were given on a 4-point scale (1 = *I never feel this way*, 4 = *I often feel this way*).

To measure *trait-perceived ostracism*, we used the Ostracism Short Scale (Rudert et al., 2020). This scale measures the perceived frequency of being ostracized within the previous 2 months and consists of four items (e.g., “Others ignored me”; Cronbach’s α = .92) answered on a 7-point scale anchored with “never” (1) to “always” (7).

#### Momentary measures

At the end of the intake survey, participants downloaded a smartphone application (ethicadata.com) through which they could access the ESM study that started on the following day. Every day, for the period of 7 days, participants received five time-triggered push notifications on their phones asking them to fill out momentary assessments. The notifications were sent randomly within the following time intervals: 9:20–11:40 (first assessment), 11:40–14:00 (second assessment), 14:00–16:20 (third assessment), 16:20–18:40 (fourth assessment), 18:40–21:00 (fifth assessment), resulting in 35 momentary assessments overall. On average, over the 1-week period, participants completed 31.09 (*SD* = 5.61) assessments (with an average of 4.59 [*SD* = 0.83] assessments per day).

All momentary measures asked about participants’ experiences within the last hour and were administered in a random order.

To measure *momentary loneliness*, participants indicated to what extent they felt lonely during the last hour (1 = *not at all*, 5 = *a great deal*).

To measure *momentary ostracism experience*, participants were asked whether during the last hour, other people (a) ignored them and (b) excluded them. Responses to these two questions were given on a 5-point scale (1 = *not at all*, 5 = *a great deal*) and averaged to measure momentary ostracism experience (*r* = .58, *p* < .001). However, the responses to both questions were severely skewed: for 90% of the assessments, participants reported not being excluded and/or ignored at all (by selecting “1 = *not at all*” on both questions) (see SOM for distribution plots). Therefore, we decided to dichotomize the momentary ostracism experience measure, with responses of 1 (*not at all*) indicating no ostracism experience (coded “0”) and responses of 2 to 5 indicating some ostracism experience (coded “1”).

Participants responded to a battery of questions about their experiences of self-control failures in the last hour. First, they indicated whether during the last hour, they have given in to a temptation: 1 = *not at all*, 5 = *a great deal*. This constituted the measure of *momentary self-control failure*. Participants who selected 2 or higher were asked additional questions. First, they indicated whether the failure was *public* (“Is anyone you know aware of what you did, for example, they saw what you did, you’ve told them, etc.”); 1 = *definitely not*, 5 = *definitely*. Second, they indicated whether this behavior (giving in to a temptation) had (a) *negative* and (b) *positive consequences for other people* (2 questions; *r* = .10, *p* < .001; 1 = *not at all*, 5 = *a great deal*). The distribution of the responses to the first (momentary self-control failure) and the last two (negative and positive consequences for others) questions was highly skewed: participants reported no self-control failures on 82% of occasions and indicated that their self-control failures had no negative or positive consequences for others on 80% and 85% of failure events, respectively; see SOM for distribution plots. We therefore decided to dichotomize these variables as well, with responses of 1 (*not at all*) indicating the absence of an event (e.g., no self-control failure, no consequences for others; coded “0”) and responses of 2 and 5 indicating the presence of an event (e.g., self-control failure, some consequences for others; coded “1”). The responses to the questions assessing the presence of others was normally distributed (see SOM) and were not transformed.

### Results

#### Self-control, perceived ostracism, and loneliness: Trait-level effects

**Figure 3.**
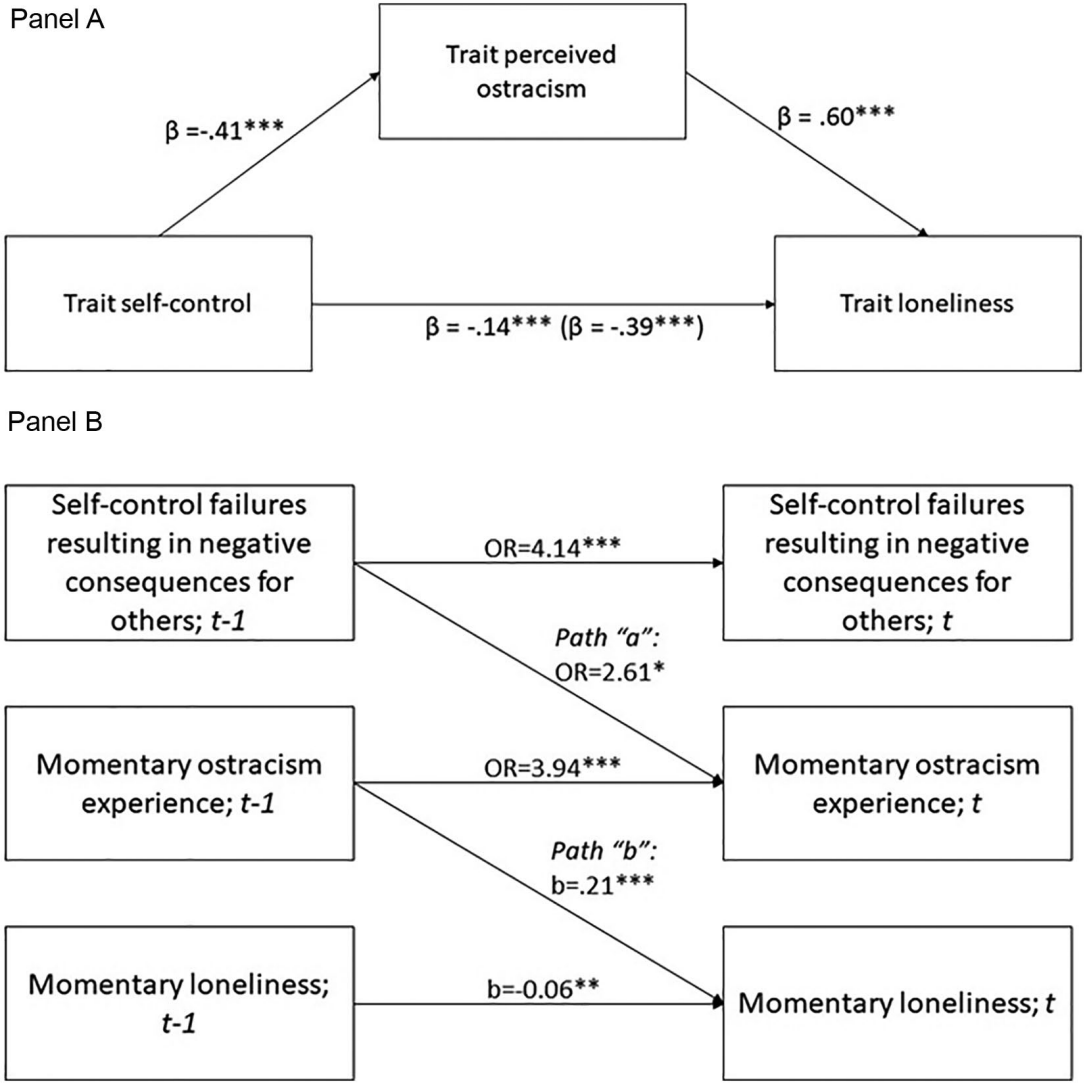
Mediation analyses (Panel A: trait measures; Panel B: longitudinal mediation, momentary assessments), Study 4. *Note.* The number inside the brackets is the total effect (c), the number outside the brackets is the direct effect (c’). *p < .05. **p < .01. ***p < .001.

Trait self-control was negatively associated with trait loneliness (*r* = −.35, *p* < .001, 95% CI = [−.45, −.25]) and trait-perceived ostracism (*r* = −.36, *p* < .001, 95% CI = [−.46, −.26]). The latter two were positively associated with each other (*r* = .63, *p* < .001, 95% CI = [.56, .70]) (see Supplemental Table S2). As expected, the effect of trait self-control on trait loneliness was mediated by trait-perceived ostracism, indirect effect −.22, *p* < .001, 95% CI = [−.24, −.21]; Figure 3 (Panel A).

#### State self-control, state ostracism, and state loneliness: Contemporaneous associations

We examined the associations between momentary self-control failures and momentary experiences of ostracism and loneliness. We used multilevel regression, with assessments nested within participants. The analyses included a random effect of participants; to account for longitudinal data structure, we additionally specified an error structure that allowed for correlations between adjacent time points for the same participant (Finch et al., 2019). We centered all continuous predictors within-persons (Enders & Tofighi, 2007).^1^ The results are presented in Table 5.

**Table 5.** Effects of Trait Self-Control and Momentary Self-Control Failures (Contemporaneous Effects), Study 4.

	DV: momentary loneliness					DV: momentary ostracism				
	Model 1	Model 2	Model 3	Model 4	Model 5	Model 1	Model 2	Model 3	Model 4	Model 5
Predictor	*b*	*b*	*b*	*b*	*b*	OR	OR	OR	OR	OR
Trait self-control	−.11**	—	—	—	—	0.69***	—	—	—	—
Momentary self-control failures	—	−.009	—	—	—	—	1.40**	—	—	—
Negative consequences	—	—	.25*	.22*	—	—	—	2.36*	2.43**	—
Positive consequences	—	—	−.05	—	−.04	—	—	0.99	—	1.03
Others’ presence	—	—	−.04^+^	−.06*	−.04	—	—	1.30**	1.36**	1.35**
Negative consequences × Others’ presence	—	—	—	.11	—	—	—	—	0.70	—
Positive consequences × Others’ presence	—	—	—	—	−.00002	—	—	—	—	0.76
N individuals	265	263	200	200	200	265	263	200	200	200
N assessments	7,717	3,347	604	604	604	7,720	3,348	604	604	604

*Note. b* = unstandardized regression coefficients; OR = odds ratios. Negative consequences = self-control failures that resulted in negative consequences for others; positive consequences = self-control failures that resulted in positive consequence for others; Others’ presence = other people’s awareness of participants’ self-control failure.

* *p* < .05. **p < .01. ***p < .001. ^+^*p* < .10.

Momentary self-control failures were associated with a higher likelihood of being ostracized (odds ratio [OR] = 1.40, *p* = .005, 95% CI = [1.11, 1.79]). In addition, self-control failures that brought about negative consequences for others were associated with a higher probability of ostracism experience (OR = 2.36, *p* = .011, 95% CI = [1.20, 4.29]).

To explore whether the effect of self-control failure is driven by instances of self-control failures with negative consequences, we created a dummy variable that distinguishes “neutral self-control failures” from the situations where no self-control failures were experienced (1 = self-control failures with neither positive nor negative consequences, 0 = no self-control failure). We regressed perceived ostracism on this new dummy. The effect of neutral (vs. no) self-control failures reached significance: OR = 1.38, *p* = .022, 95% CI = [1.05, 1.80]). Hence, even self-control failures without positive or negative consequences for others were associated with more perceived ostracism (relative to no self-control failures).

Momentary self-control failures were not significantly associated with momentary loneliness (*b* = −0.01, *p* = .74). However, self-control failures that had negative consequences for others were positively associated with feeling lonely (*b* = 0.25, *p* = .015, 95% CI = [0.04, 0.43]), see Table 5.

#### State self-control, state ostracism, and state loneliness: Prospective effects and longitudinal mediation

We assessed prospective effects of self-control failures and self-control failures with negative consequences for others on loneliness and perceived ostracism, and another way around. We computed the lagged values of these variables for each individual and each day and used the same multilevel analysis approach as descried above. The results are shown in Table 6. Self-control failures were not prospectively associated with either perceived ostracism or loneliness. However, experiencing a self-control failure with negative consequences for others predicted feeling ostracized (OR = 2.61, *p* = .023, 95% CI = [1.15, 5.99]) and lonely (*b* = 0.39, *p* = .001, 95% CI = [0.17, 0.61]) over time.

**Table 6.** Momentary Assessments: Prospective Effects, Study 4.

Model	Predictor	DV: loneliness at *t* (*b*)	DV: ostracism at *t* (OR)	DV: self-control failure at *t* (OR)	DV: negative consequences at *t* (OR)
Model 1	Loneliness at *t−*1	−.04^+^	—	**0.84** ^+^	—
	Self-control failure at *t−*1	**.02**	—	0.95	—
	*N* (persons/assessments)	259/2,571	—	259/2,569	—
Model 2	Loneliness at *t−*1	−.02	—	—	**1.04**
	Negative consequences at *t−*1	**.39** ***	—	—	1.82***
	*N* (persons/assessments)	179/442	—	—	71/119
Model 3	Ostracism at *t−*1	—	1.82***	**0.98**	—
	Self-control failure at *t−*1	—	**1.02**	0.96	—
	*N* (persons/assessments)	—	259/2,573	259/2,570	—
Model 4	Ostracism at *t−*1	—	3.94***	—	**1.23** **
	Negative consequences at *t−*1	—	**2.61** *	—	1.70***
	*N* (persons/assessments)	—	179/442	—	71/119

*Note. b* = unstandardized regression coefficients; OR = odds ratios. Negative consequences = self-control failures that resulted in negative consequences for others; positive consequences = self-control failures that resulted in positive consequence for others; Others’ presence = other people’s awareness of participants’ self-control failure. Cross-lagged effects are in bold.

* *p* < .05. **p < .01. ***p < .001. +p < .10.

Neither feeling lonely nor ostracized was associated with the likelihood of committing self-control failures at a later time point; however, perceived ostracism (but not loneliness) was prospectively associated with a higher likelihood of engaging in self-control failures with negative consequences for others (OR = 1.23, *p* = .005, 95% CI = [1.07, 1.42]).

We tested whether perceived ostracism mediates the effect of self-control failures with negative consequences for others on participants’ loneliness. We conducted longitudinal mediation analysis by estimating prospective paths “a” and “b” (Cole & Maxwell, 2003; Preacher, 2015): we regressed perceived ostracism at *t* on self-control failures with negative consequences at *t*−1 (path “a”) and perceived ostracism at *t−*1; and we regressed loneliness at *t* on perceived ostracism at *t−*1 (path “b”) and loneliness at *t−*1. We used the Monte Carlo simulation method to estimate the significance of the indirect effect (Selig & Preacher, 2008). The results are shown in Figure 3: self-control failures with negative consequences for others predicted increased probability of ostracism, which in turn predicted higher loneliness over time. The indirect effect was significant (.20, 95% CI = [.03; .41]).

### Discussion

Studies 1 to 3 showed that people with lower (trait and state) self-control tend to report higher loneliness and elicit stronger ostracism intentions from others. Study 4 complemented these findings by demonstrating that low self-control individuals’ increased ostracism experience is likely to explain their elevated feelings of loneliness. It also highlighted the importance of other-harming consequences: while self-control failures were generally associated with more perceived ostracism, only self-control failures that brought about negative consequences for others were associated with increased feelings of loneliness.

Finally, other-harming self-control failures predicted increased ostracism and loneliness over time and experiences of ostracism (but not loneliness) predicted committing more other-harming self-control failures over time, providing some evidence for bidirectional effects.

## General Discussion

Across four studies (and three additional studies reported in SOM) using correlational, experimental, daily diary, and ESM methods, we found evidence for low self-control being a risk factor for increased loneliness.

Trait self-control predicted less chronic (trait) loneliness in a large nationally representative data set from the Netherlands (Study 1) and less experience of loneliness in everyday life in samples of American (Study 2) and British (Study 4) adults. Not only trait but also state self-control (daily self-control failures) was associated with everyday loneliness (Study 2). Longitudinal analyses (Study 2) established temporal precedence of self-control by showing that self-control failures on one day predicted increased loneliness on the following day.

Why is low self-control associated with more loneliness? Given the importance of self-regulation for interpersonal success (Baumeister & Exline, 1999; Baumeister & Vohs, 2007), we proposed that self-control could lead to less loneliness as it helps to prevent social exclusion. Self-control is crucial for overcoming selfish impulses and is vital for group survival (Baumeister & Vohs, 2007). Self-control facilitates norm-compliance (DeBono et al., 2011), faithfulness in romantic relationships (Pronk et al., 2011), and cooperative behaviors in economic games (Kocher et al., 2017). Therefore, in social situations, individuals might prefer high self-control others as interaction partners and exclude individuals who seem to lack self-control. Indeed, Study 3 showed that people are more likely to see others who fail (vs. succeed) at self-control as being less able to behave in accordance with prosocial norms and are therefore more willing to ostracize them. Study 4’s ESM study has further underscored the role of ostracism in the relationship between self-control and loneliness. It showed that momentary self-control failures were associated with momentary experiences of ostracism. Importantly, a longitudinal mediation analysis showed that self-control failures resulting in negative consequences for others were prospectively associated with increased ostracism experience and, consequently, higher loneliness.

While some self-control failures are harmful only for the self (e.g., overspending), other self-control failures bring about negative consequences for others (e.g., free-riding). Do self-control failures elicit ostracism and lead to loneliness only when they are harmful to others? Our results provide some indication that the presence of negative consequences for others is not necessary for self-control lapses to lead to ostracism but seems to be important to elicit loneliness. In Study 4, participants reported to feel ostracized by others following any self-control failure; in contrast, only self-control failures resulting in negative consequences for others were associated with loneliness. Similarly, in Study S1 (reported in SOM), participants believed that not sticking to their diet (a self-control failure that is presumably harmless to others) would make others socially exclude them without making them feel lonely. We speculate that since loneliness is rooted in social experiences (or the absence thereof), it is more likely to arise when individuals’ actions have repercussions for others. Further replications of this pattern are needed to confirm this interpretation.

Even though the association between trait self-control and loneliness emerged consistently across studies, the effect size was small (judging by standard measures of effect sizes). It is still noteworthy that it was similar in size to the effects of established predictors of loneliness, such as agreeableness and extraversion (Mund & Neyer, 2016). However, compared to trait self-control, the effect of state self-control appeared much higher in magnitude. For example, committing a self-control failure was associated with 42% higher risk of social exclusion (and committing an other-harming self-control failure was associated with more than two times higher risks of exclusion; Study 4).

Can the associations between self-control and perceived ostracism be explained by low self-control people misperceiving others’ behavior toward them as more hostile than it actually is? Study 3 provides some evidence against this alternative explanation: it demonstrated that observers show a stronger ostracism intention toward a low (vs. a high) self-control target. Nevertheless, we acknowledge that the use of a hypothetical scenario in Study 3 has limitations, and we encourage future studies to explore whether observing others’ self-control failure translates into actual ostracism behavior.

On a related note, even though participants were more willing to ostracize a low than a high self-control target, the overall level of ostracism intentions was low (2.34 on a 7-point scale). The reluctance to engage in ostracism has been reported in other studies using similar measures and can be potentially explained by inclusion (rather than exclusion) being the most common behavior in social interactions (Ren & Evans, 2020; Rudert & Greifeneder, 2016).

The present findings contribute to several research areas. They extend the literature on the social consequences of self-control. Several studies demonstrated that lacking self-control could be a risk factor for existing relationships: low self-control individuals demonstrate less forgiveness and stronger retaliation intentions (Burnette et al., 2014), are more prone to aggression (Wilkowski et al., 2010), are less likely to stay faithful to their romantic partners (Pronk et al., 2011), and report a lower relationship quality (Vohs et al., 2011). The present investigation contributes to this literature by showing that low self-control might not only damage existing relationships but also prevent people from getting into relationships in the first place.

The present research adds to the effort directed at the integration of the literatures on trait and state self-control that have had little cross-talk so far (de Ridder et al., 2018). Previous research defined state self-control as a state of resource depletion following an effortful inhibition task and did not consistently link it to trait self-control. In contrast, following Fleeson (2001), we operationalized state self-control as a behavioral manifestation of trait self-control and found consistently positive interrelations between the two, contributing to bridging the gap between trait and state self-control research.

While the present studies provided compelling evidence for one specific mechanism through which low self-control can contribute to loneliness, there might be several other mechanisms worth exploring. For example, it is possible that low self-control individuals feel lonely not only because other people tend to ostracize them but also because they themselves prefer to withdraw from social interactions. Consistent with this idea, Delelis and Christophe (2018) have shown that people tend to seek isolation after a negative emotional episode (and one’s failure to resist a temptation could be considered one). In contrast, de Hooge et al. (2018) showed that some negative emotional experiences—that is, experimentally induced shame—result in weaker social withdrawal tendencies.

Two additional preregistered experiments (Study S2 and S3, reported in SOM) tested this alternative possibility. We experimentally manipulated self-control by randomly assigning participants to recall a time where they failed (vs. succeeded) at resisting a temptation. After the manipulation, we measured participants’ intention to withdraw from social interactions (e.g., “Reliving the experience I wrote about makes me want to stay alone and speak to no one.”). Although the manipulation was successful, in none of these additional studies (overall *N* = 449) did it significantly affect social withdrawal intentions (*p* values > .10). However, as the absence of a significant effect might be a result of our reliance on a specific manipulation of self-control failure, more studies are needed before this alternative explanation can be ruled out.

The present findings point at multiple avenues for future investigations. For example, Study 3 showed that to social observers, self-control might signal the ability to behave in accordance with the prosocial norms. Yet, sometimes group norms dictate antisocial or even criminal behaviors, such as cheating or deception. A potentially interesting question to future research is to explore whether people ascribe high (vs. low) self-control others a greater ability to behave in accordance with such antisocial norms too and whether, in this case, high self-control would backfire and result in ostracism and loneliness (Kokkoris & Stavrova, 2020). In a similar vein, herein we focused on self-control failures that imply negative consequences for others (Study 4). However, sometimes, behaviors that represent self-control failures for an individual might be encouraged and approved by others (e.g., alcohol overconsumption at parties). We assume that such socially encouraged self-control failures would not result in exclusion and loneliness; yet it remains to be explored in future studies.

The large-scale national survey, one experiment and two intensive longitudinal (7-day long) studies provided robust evidence of the effect of self-control on loneliness in the short run. We hope that future studies will explore whether the effect of self-control on loneliness persists in the long run (e.g., across decades) and whether it affects loneliness development across the life span.

Using longitudinal data, we have shown that self-control failures prospectively predict loneliness, while loneliness does not prospectively predict self-control failures (Studies 2 and, partially, 4). Yet, individuals who felt ostracized at one point in time were more likely to commit self-control failures harming others at the following time point (Study 4). This finding extends some past experimental work showing that social exclusion interferes with one’s self-regulatory ability, resulting in unhealthy food consumption and attention deficits (Baumeister et al., 2005; Campbell et al., 2006). However, more experimental and longitudinal studies are needed to fully understand whether the potential bidirectional relationships between social exclusion and self-control failures.

Finally, the present findings might have implications for whether and how people communicate their self-control experiences to others. As people show a stronger willingness to ostracize low self-control others (Study 3) and anticipate to get ostracized themselves in case of a self-control failure (Study S1), we speculate that people might be rather unwilling to let other people know about their failures at resisting temptations. At the same time, relationship research suggests that sharing personal information and making oneself vulnerable might represent an important building block of intimacy in interpersonal relationships (Collins & Miller, 1994). Hence, we hope future studies would show whether people tend to not disclose their personal self-control failures and to what extent this represents a viable strategy for reaching inclusion and acceptance versus exclusion and alienation.

## Supplemental Material

sj-docx-1-psp-10.1177_01461672211007228 – Supplemental material for Low Self-Control: A Hidden Cause of Loneliness? Click here for additional data file. Supplemental material, sj-docx-1-psp-10.1177_01461672211007228 for Low Self-Control: A Hidden Cause of Loneliness? by Olga Stavrova, Dongning Ren and Tila Pronk in Personality and Social Psychology Bulletin

## Notes

1 In case of binary predictors, within-person centered coefficients would reflect the effect of experiencing an event (e.g., self-control failure) depending on how often that participant has experienced the event during the study period. As we were not interested in this question, we decided not to center binary predictors.
