# Supplementary material for: Low Self-Control: A Hidden Cause of Loneliness?
Source: Pers Soc Psychol Bull. 2021 Apr 15;48(3):347–62. doi: 10.1177/01461672211007228 (PMC8855382; doi:10.1177/01461672211007228)
Supplement: sj-docx-1-psp-10.1177_01461672211007228 – Supplemental material for Low Self-Control: A Hidden Cause of Loneliness? [file sj-docx-1-psp-10.1177_01461672211007228.docx]

**Materials**

**Study 1**

Self-control

**gg12a034 – gg12a046**

The next statements are about how you feel about yourself. Please indicate to what extent the statements are applicable to you.

**gg12a034** I am good at resisting temptation.

**gg12a035** I have a hard time breaking bad habits.

**gg12a036** I am lazy.

**gg12a037** I say inappropriate things.

**gg12a038** I do certain things that are bad for me, if they are fun.

**gg12a039** I refuse things that are bad for me.

**gg12a040** I wish I had more self-discipline.

**gg12a041** People would say that I have iron self- discipline.

**gg12a042** Pleasure and fun sometimes keep me from getting work done.

**gg12a043** I have trouble concentrating.

**gg12a044** I am able to work effectively toward long-term goals.

**gg12a045** Sometimes I can’t stop myself from doing something, even if I know it is wrong.

**gg12a046** I often act without thinking through all the alternatives.

Loneliness

Can you indicate for each statement to what degree it applies to you, based on how you

are feeling at present?

**cs12e284** I have a sense of emptiness around me

**cs12e285** there are enough people I can count on in case of a misfortune

**cs12e286** I know a lot of people that I can fully rely on

**cs12e287** there are enough people to whom I feel closely connected

**cs12e288** I miss having people around me

**cs12e289** I often feel deserted

1 yes

2 no

3 don’t know/don’t want to say

Big Five

On the following pages, there are phrases describing people's behaviors. Please use the rating scale below to describe how accurately each statement describes ***you***. Describe yourself as you generally are now, not as you wish to be in the future. Describe yourself as you honestly see yourself, in relation to other people you know of the same sex as you are, and roughly your same age. Please read each statement carefully, and then fill in the bubble that corresponds to the number on the scale.

1 = very inaccurate

2 = moderately inaccurate

3 = neither inaccurate nor accurate 4 = moderately accurate

5 = very accurate

**cp12e020 - cp12e029**

Please use the rating scale below to describe how accurately each statement describes ***you***.

**cp12e020** Am the life of the party.

**cp12e021** Feel little concern for others.

**cp12e022** Am always prepared.

**cp12e023** Get stressed out easily.

**cp12e024** Have a rich vocabulary.

**cp12e025** Don’t talk a lot.

**cp12e026** Am interested in people.

**cp12e027** Leave my belongings around.

**cp12e028** Am relaxed most of the time.

**cp12e029** Have difficulty understanding abstract ideas.

1 very inaccurate

2 moderately inaccurate

3 neither inaccurate nor accurate

4 moderately accurate

5 very accurate

**cp12e030 - cp12e039**

Please use the rating scale below to describe how accurately each statement describes ***you***.

**cp12e030** Feel comfortable around people.

**cp12e031** Insult people.

**cp12e032** Pay attention to details.

**cp12e033** Worry about things.

**cp12e034** Have a vivid imagination.

**cp12e035** Keep in the background.

**cp12e036** Sympathize with others’ feelings.

**cp12e037** Make a mess of things.

**cp12e038** Seldom feel blue**.**

**cp12e039** Am not interested in abstract ideas.

1 very inaccurate

2 moderately inaccurate

3 neither inaccurate nor accurate

4 moderately accurate

5 very accurate

**cp12e040 - cp12e049**

Please use the rating scale below to describe how accurately each statement describes ***you***.

**cp12e040** Start conversations.

**cp12e041** Am not interested in other people’s problems.

**cp12e042** Get chores done right away.

**cp12e043** Am easily disturbed.

**cp12e044** Have excellent ideas.

**cp12e045** Have little to say.

**cp12e046** Have a soft heart.

**cp12e047** Often forget to put things back in their proper place.

**cp12e048** Get upset easily.

**cp12e049** Do not have a good imagination.

1 very inaccurate

2 moderately inaccurate

3 neither inaccurate nor accurate

4 moderately accurate

5 very accurate

**cp12e050 - cp12e059**

Please use the rating scale below to describe how accurately each statement describes ***you***.

**cp12e050** Talk to a lot of different people at parties.

**cp12e051** Am not really interested in others.

**cp12e052** Like order.

**cp12e053** Change my mood a lot.

**cp12e054** Am quick to understand things.

**cp12e055** Don’t like to draw attention to myself.

**cp12e056** Take time out for others.

**cp12e057** Shirk my duties.

**cp12e058** Have frequent mood swings.

**cp12e059** Use difficult words.

1 very inaccurate

2 moderately inaccurate

3 neither inaccurate nor accurate

4 moderately accurate

5 very accurate

**cp12e060 - cp12e069**

Please use the rating scale below to describe how accurately each statement describes ***you***.

**cp12e060** Don’t mind being the center of attention.

**cp12e061** Feel others’ emotions.

**cp12e062** Follow a schedule.

**cp12e063** Get irritated easily.

**cp12e064** Spend time reflecting on things.

**cp12e065** Am quiet around strangers.

**cp12e066** Make people feel at ease.

**cp12e067** Am exacting in my work.

**cp12e068** Often feel blue.

**cp12e069** Am full of ideas.

1 very inaccurate

2 moderately inaccurate

3 neither inaccurate nor accurate

4 moderately accurate

5 very accurate

**Study 2**

Trait measures

Meaning in life

Please take a moment to think about what makes your life feel important to you. Please respond to the following statements as truthfully and accurately as you can, and also please remember that these are very subjective questions and that there are no right or wrong answers.

|  | Absolutely Untrue (1) | Mostly Untrue (2) | Somewhat Untrue (3) | Can’t Say True or False (4) | Somewhat True (5) | Mostly True (6) | Absolutely True (7) |
| --- | --- | --- | --- | --- | --- | --- | --- |
| I understand my life’s meaning. (1) |  |  |  |  |  |  |  |
| I am looking for something that makes my life feel meaningful. (2) |  |  |  |  |  |  |  |
| I am always looking to find my life’s purpose. (3) |  |  |  |  |  |  |  |
| My life has a clear sense of purpose. (4) |  |  |  |  |  |  |  |
| I have a good sense of what makes my life meaningful. (5) |  |  |  |  |  |  |  |
| I have discovered a satisfying life purpose. (6) |  |  |  |  |  |  |  |
| I am always searching for something that makes my life feel significant. (7) |  |  |  |  |  |  |  |
| I am seeking a purpose or mission for my life. (8) |  |  |  |  |  |  |  |
| My life has no clear purpose. (9) |  |  |  |  |  |  |  |
| I am searching for meaning in my life. (10) |  |  |  |  |  |  |  |

Self-control

Using the scale provided, please indicate how much each of the following statements reflects how you typically are.

|  | Not at all (1) | (2) | (3) | (4) | Very much (5) |
| --- | --- | --- | --- | --- | --- |
| I am good at resisting temptation. (1) |  |  |  |  |  |
| I have a hard time breaking bad habits. (2) |  |  |  |  |  |
| I am lazy. (3) |  |  |  |  |  |
| I say inappropriate things. (4) |  |  |  |  |  |
| I do certain things that are bad for me, if they are fun. (5) |  |  |  |  |  |
| I refuse things that are bad for me. (6) |  |  |  |  |  |
| I wish I had more self-discipline. (7) |  |  |  |  |  |
| People would say that I have iron self- discipline. (8) |  |  |  |  |  |
| Pleasure and fun sometimes keep me from getting work done. (9) |  |  |  |  |  |
| I have trouble concentrating. (11) |  |  |  |  |  |
| I am able to work effectively toward long-term goals. (13) |  |  |  |  |  |
| Sometimes I can’t stop myself from doing something, even if I know it is wrong. (14) |  |  |  |  |  |
| I often act without thinking through all the alternatives. (15) |  |  |  |  |  |

Life satisfaction

Taking all things together, how satisfied are you with your life as a whole?

|  | Extremely dissatisfied (1) | (2) | (3) | (4) | (5) | (6) | (7) | (8) | (9) | Extremely satisfied (10) |
| --- | --- | --- | --- | --- | --- | --- | --- | --- | --- | --- |
| (1) |  |  |  |  |  |  |  |  |  |  |

Daily measures

In the past 24 hours, did any of the following happen to you?

|  | Not at all (1) | (2) | (3) | (4) | (5) | (6) | A lot (7) |
| --- | --- | --- | --- | --- | --- | --- | --- |
| You felt lonely? (49) |  |  |  |  |  |  |  |
| You felt that your life was meaningful? (53) |  |  |  |  |  |  |  |
| You gave in to a temptation? (55) |  |  |  |  |  |  |  |
| You felt happy? (56) |  |  |  |  |  |  |  |
| You felt sad? (57) |  |  |  |  |  |  |  |

**Study 3**

Self-control non-failure condition

In this study, we will ask you to make several judgments about Robin, an Mturk worker.

Robin is 30 year old, works full-time and has a three-year old daughter.
Robin took part in one of our previous studies where we asked participants to describe an event that had happened to them in the previous week.

Below you will see Robin's response.

*Lately I have some money problems . . . it’s not that I’m in debt, but I definitely need to save some money for my further education which is really important for me! Last Wednesday I was around the city having a walk, and I ended up in my favorite electronics store. I was having a look at all the cool smartphones and tablets available and at the new entries, but eventually I did not buy anything.*

Self-control failure condition

In this study, we will ask you to make several judgments about Robin, an Mturk worker.

Robin is 30 year old, works full-time and has a three-year old daughter.
Robin took part in one of our previous studies where we asked participants to describe an event that had happened to them in the previous week.

Below you will see Robin's response.


*Lately I have some money problems . . . it’s not that I’m in debt, but I definitely need to save some money for my further education which is really important for me! Last Wednesday I was around the city having a walk, and I ended up in my favorite electronics store. I was having a look at all the cool smartphones and tablets available and at the new entries, and I ended up buying a new smartphone (even though I already had one).*

Ostracism intentions

Imagine that Robin is a new colleague at your work and indicate to what extent you agree or disagree with the following statements.

|  | Strongly disagree (1) | Disagree (2) | Somewhat disagree (3) | Neither agree nor disagree (4) | Somewhat agree (5) | Agree (6) | Strongly agree (7) |
| --- | --- | --- | --- | --- | --- | --- | --- |
| I would invite Robin to events with our group of friends. (1) |  |  |  |  |  |  |  |
| I might find myself ignoring Robin. (2) |  |  |  |  |  |  |  |
| I would want to encourage other people to ignore Robin. (3) |  |  |  |  |  |  |  |
| I might consider turning my back on Robin. (4) |  |  |  |  |  |  |  |
| I might feel the urge to give Robin the silent treatment. (6) |  |  |  |  |  |  |  |
| I might find myself excluding Robin. (7) |  |  |  |  |  |  |  |

Motivation to behave prosocially

Indicate to what extent you agree or disagree with the following statements.

|  | Strongly disagree (1) | Disagree (2) | Somewhat disagree (3) | Neither agree nor disagree (4) | Somewhat agree (5) | Agree (6) | Strongly agree (7) |
| --- | --- | --- | --- | --- | --- | --- | --- |
| Robin cares about other people. (1) |  |  |  |  |  |  |  |
| Robin takes time for others. (2) |  |  |  |  |  |  |  |
| Robin sympathizes with others’ feelings. (3) |  |  |  |  |  |  |  |

Ability to behave prosocially

Indicate to what extent you agree or disagree with the following statements.

|  | Strongly disagree (1) | Disagree (2) | Somewhat disagree (3) | Neither agree nor disagree (4) | Somewhat agree (5) | Agree (6) | Strongly agree (7) |
| --- | --- | --- | --- | --- | --- | --- | --- |
| Robin has enough will power to not engage in behaviors that might hurt others. (1) |  |  |  |  |  |  |  |
| Robin is able to resist the temptation to behave selfishly. (2) |  |  |  |  |  |  |  |
| Robin can easily follow the socially desirable and acceptable standards of behavior. (3) |  |  |  |  |  |  |  |

**Study 4**

Trait measures

Indicate how often each of the statements below is descriptive of you.

|  | I never feel this way (1) | I rarely feel this way (2) | I sometimes feel this way (3) | I often feel this way (4) |
| --- | --- | --- | --- | --- |
| I am unhappy doing so many things alone (1) |  |  |  |  |
| I have nobody to talk to (2) |  |  |  |  |
| I cannot tolerate being so alone (3) |  |  |  |  |
| I lack companionship (4) |  |  |  |  |
| I feel as if nobody really understands me (5) |  |  |  |  |
| I find myself waiting for people to call or write (6) |  |  |  |  |
| There is no one I can turn to (7) |  |  |  |  |
| I am no longer close to anyone (8) |  |  |  |  |
| My interests and ideas are not shared by those around me (9) |  |  |  |  |
| I feel left out (10) |  |  |  |  |
| I feel completely alone (11) |  |  |  |  |
| I am unable to reach out and communicate with those around me (12) |  |  |  |  |
| My social relationships are superficial (13) |  |  |  |  |
| I feel starved for company (14) |  |  |  |  |
| No one really knows me well (15) |  |  |  |  |
| I feel isolated from others (16) |  |  |  |  |
| I am unhappy being so withdrawn (17) |  |  |  |  |
| It is difficult for me to make friends (18) |  |  |  |  |
| I feel shut out and excluded by others (19) |  |  |  |  |
| People are around me but not with me (20) |  |  |  |  |

Q60 Using the scale provided, please indicate how much each of the following statements reflects how you typically are.

|  | Not at all (1) | (2) | (3) | (4) | Very much (5) |
| --- | --- | --- | --- | --- | --- |
| I am good at resisting temptation. (1) |  |  |  |  |  |
| I have a hard time breaking bad habits. (2) |  |  |  |  |  |
| I am lazy. (3) |  |  |  |  |  |
| I say inappropriate things. (4) |  |  |  |  |  |
| I do certain things that are bad for me, if they are fun. (5) |  |  |  |  |  |
| I refuse things that are bad for me. (6) |  |  |  |  |  |
| I wish I had more self-discipline. (7) |  |  |  |  |  |
| People would say that I have iron self- discipline. (8) |  |  |  |  |  |
| Pleasure and fun sometimes keep me from getting work done. (9) |  |  |  |  |  |
| To monitor data quality, please select the middle of the scale here. (10) |  |  |  |  |  |
| I have trouble concentrating. (11) |  |  |  |  |  |
| I am able to work effectively toward long-term goals. (13) |  |  |  |  |  |
| Sometimes I can’t stop myself from doing something, even if I know it is wrong. (14) |  |  |  |  |  |
| I often act without thinking through all the alternatives. (15) |  |  |  |  |  |

Q98 How often did you experience the following occurrences during the last two months?

|  | Never (1) | (2) | (3) | (4) | (5) | (6) | Always (7) |
| --- | --- | --- | --- | --- | --- | --- | --- |
| Others ignored me (1) |  |  |  |  |  |  |  |
| Others shut me out from the conversation (2) |  |  |  |  |  |  |  |
| Others treated me as if I wasn’t there (3) |  |  |  |  |  |  |  |
| Others did not invite me to activities (4) |  |  |  |  |  |  |  |

Q77
Please provide an answer for each of the following questions. 


Please take the time to answer these questions seriously. Here's why, we test whether you actually take the time to read the instructions. 


Therefore, if you read this, please answer 'three' on the first question, and 'five' on the second and third question. 


These questions give us important background information about the participants.

|  | Not at all  1 (1) | 2 (2) | 3 (3) | 4 (4) | 5 (5) | 6 (6) | 7 (7) | 8 (8) | Very much  9 (9) |
| --- | --- | --- | --- | --- | --- | --- | --- | --- | --- |
| I prefer to get bonuses over steady income (1) |  |  |  |  |  |  |  |  |  |
| I think receiving a bonus within 2 weeks is fast enough (2) |  |  |  |  |  |  |  |  |  |
| I prefer experiments where I can interact with other people (3) |  |  |  |  |  |  |  |  |  |

Momentary measures

| (Q#59): | During the last hour, to what extent have you felt **lonely**?   \| 1) \| ○ \| not at all \| \| --- \| --- \| --- \| \| 2) \| ○ \| a little \| \| 3) \| ○ \| a moderate amount \| \| 4) \| ○ \| a lot \| \| 5) \| ○ \| a great deal \| |
| --- | --- | --- | --- | --- | --- | --- | --- | --- | --- | --- | --- | --- | --- | --- | --- | --- |

| (Q#114): | During the last hour, have you given in to a temptation?   \| 1) \| ○ \| not at all \| \| --- \| --- \| --- \| \| 2) \| ○ \| a little \| \| 3) \| ○ \| a moderate amount \| \| 4) \| ○ \| a lot \| \| 5) \| ○ \| a great deal \| |
| --- | --- | --- | --- | --- | --- | --- | --- | --- | --- | --- | --- | --- | --- | --- | --- | --- |

| (Q#67): | Is anyone you know aware of what you did (e.g., they saw what you did, you’ve told them, etc.)?   \| 1) \| ○ \| definitely not \| \| --- \| --- \| --- \| \| 2) \| ○ \| probably not \| \| 3) \| ○ \| probably \| \| 4) \| ○ \| very probably \| \| 5) \| ○ \| definitely \| |
| --- | --- | --- | --- | --- | --- | --- | --- | --- | --- | --- | --- | --- | --- | --- | --- | --- |

| (Q#66): | Does this behavior (giving in to a temptation) have **negative consequences for other people**?   \| 1) \| ○ \| not at all \| \| --- \| --- \| --- \| \| 2) \| ○ \| a little \| \| 3) \| ○ \| a moderate amount \| \| 4) \| ○ \| a lot \| \| 5) \| ○ \| a great deal \| |
| --- | --- | --- | --- | --- | --- | --- | --- | --- | --- | --- | --- | --- | --- | --- | --- | --- |

| (Q#69): | Does this behavior (giving in to a temptation) have **positive consequences for other people**?   \| 1) \| ○ \| not at all \| \| --- \| --- \| --- \| \| 2) \| ○ \| a little \| \| 3) \| ○ \| a moderate amount \| \| 4) \| ○ \| a lot \| \| 5) \| ○ \| a great deal \| |
| --- | --- | --- | --- | --- | --- | --- | --- | --- | --- | --- | --- | --- | --- | --- | --- | --- |

| (Q#103): | During the last hour, **did other people ignore you?**   \| 1) \| ○ \| not at all \| \| --- \| --- \| --- \| \| 2) \| ○ \| a little \| \| 3) \| ○ \| a moderate amount \| \| 4) \| ○ \| a lot \| \| 5) \| ○ \| a great deal \| |
| --- | --- | --- | --- | --- | --- | --- | --- | --- | --- | --- | --- | --- | --- | --- | --- | --- |

| (Q#104): | During the last hour, **did other people exclude you?**   \| 1) \| ○ \| not at all \| \| --- \| --- \| --- \| \| 2) \| ○ \| a little \| \| 3) \| ○ \| a moderate amount \| \| 4) \| ○ \| a lot \| \| 5) \| ○ \| a great deal \| |
| --- | --- | --- | --- | --- | --- | --- | --- | --- | --- | --- | --- | --- | --- | --- | --- | --- |
